# Globalisation of birth markets: a case study of assisted reproductive technologies in India

**DOI:** 10.1186/1744-8603-7-27

**Published:** 2011-08-12

**Authors:** Nadimpally Sarojini, Vrinda Marwah, Anjali Shenoi

**Affiliations:** 1 Sama-Resource Group for Women and Health, B-45 2nd floor, Shivalik Main Road, Malviya Nagar, New Delhi 110017, India

## Abstract

The escalation of Assisted Reproductive Technologies (ARTs) in India into a veritable fertility industry is the result of a multitude of reasons. This paper places the bio-genetic industry within the larger political economy framework of globalisation and privatisation, thus employing a framework that is often omitted from discussions on ARTs, but has direct and significant bearings on the ART industry in India. As markets for human organs, tissues and reproductive body parts experience unprecedented growth, the limits of what can or should be bought and sold continue to be pushed. As such, bodies have emerged as sale-worthy economic capital. Commercial flows of reproductive material create and deploy the division of the body into parts over which ownership is claimed, in the process following 'modern routes of capital' and raising issues of structural inequality.

This paper presents a brief picture of India's fertility industry with specific focus on its ground-level operation, nature and growth. It aims to explore the industry dimensions of ARTs, by highlighting the macro picture of health care markets and medical tourism in India, the proliferation of the ART industry, market features such as the social imperative to mother, costs, promotion and marketing, unverified claims, inflated success rates, deals and offers, actors and collaborations in the field, and finally, the absence of standards. This paper presents findings from the research 'Constructing Conceptions: The Mapping of Assisted Reproductive Technologies in India', by Sama, a Delhi-based resource group working on gender, health and rights. This research was conducted from 2008 to 2010 in the three states of Uttar Pradesh, Orissa and Tamil Nadu in India, and is one of the first of its kind, highlighting unethical medical practices and making a case for the regulation of the ART industry. As such, it forms a significant part of Sama's ongoing work on women and technologies, particularly policy-level advocacy.

## 1. Introduction: The contours of biogenetic trade

The advent of new genetic technologies and the policies of privatisation corresponding to globalisation are not independent of one another. Biotechnology is at once promoted by the state as the high-technology answer to, amongst others, the hollowing-out effects of globalisation, and is celebrated as a 'cutting edge contribution' to health care. Yet a case is made that these technologies, though beneficial, cannot be provided in the public health set up. This further compounds their 'need' and proliferation in the private sector, thus chipping away at the already withering welfare state; only a few states provide these technologies in their public health systems, in a bid to increase their populations. As such, for most part, it is the rich who continue to be vested with a set of privileges. Foucault, among others, has described this privileging, or this flow of power, as bio-power, operating in and through the significant historical transition contemporaneous with the shaping of industrial capitalism, in which emphasis shifts from the primacy of sovereignty, law, and coercion-- or the force "to take life"-- to the development of new forms of power constitutive *of *life [1]. This differential access can be understood as 'stratified reproduction' (a term coined by Ginsburg and Rapp) [2], an unequal power equation by which some categories of people are encouraged or empowered to reproduce, while reproduction in others is devalued. It is clear that in a climate of state restructuring and privatisation, the priorities of the state in relation to health care have shifted from protecting the public good to promoting the interests of industry, thus creating the conditions for health care to be 'a site of corporate profit' [3]

Commerce and economics are often omitted from discussions and debates on ARTs and related genetic-biotech issues. However, this is a significant omission. As anthropologist Kaushik Sunder Rajan has written in his study of Biocapital: "One can understand emergent biotechnologies such as genomics only by simultaneously analyzing the market frameworks within which they emerge" [4]. Any careful study of the operationalization of the ART industry would do well to undertake a political economy focus, in order to critically and holistically situate this phenomenon in the context of globalisation and state restructuring. Though often under-researched, financial markets make up the framework within which ARTs and related biotechnologies are flourishing today. As Kean Birch, points out," the biosciences rely on a future-oriented market that enables the generation of short-term value (i.e., in shares or venture capital returns) on the back of expectations that there is then no necessity to fulfil" [5].

This is true also for human reproduction. Scheper-Hughes quotes George Soros who asserts that markets are "indiscriminate [and] promiscuous... [and] reduce everything, including human beings and their sexual and reproductive capacities to the status of commodities, things [that] can be bought, sold, traded and stolen" [6]. While the commodification of the human body may not be new, the explosion in markets for human organs, tissues and reproductive body parts that we are witnessing today is unprecedented. In the contemporary moment, the boundary between what can or cannot, and should or should not, be bought and sold has been blurred. A cursory look at the current markets for human body parts makes this clear. While the sale of organs is illegal in most countries, semen, ova, blood and other body fluids and tissues fall outside the purview of existing legislations because of their regenerative nature [7]. Thus, bodies have emerged as sale-worthy economic capital. Biomedicine and biotechnology are the foremost sites where new technologies have been created to fragment body parts, giving them an existence outside of the human body, allowing them to be exchanged for compensation or commercial transaction, and thus making them resources in their own right. As Sharp puts it, biomedicine has "quickly fragmented [the body] and transformed [it] into scientific work projects" [8]. While reproductive materials and organs have, on the one hand, assumed an independent and individualised existence and have become the private property of the person selling them, on the other hand, the physical, social and cultural attributes of the donor affect the price of the reproductive material. Therefore, both objectification and personification are parallel processes at play here [9]. Further, the movement of reproductive material and processes follows along "modern routes of capital" flow - from "South to North, from third world to first world, from poor to rich bodies, from black and brown to white bodies, from young to old bodies, from productive to less productive...bodies"[10]. It is these processes and structures, which trade in reproductive material operates within and through, that raise significant questions for theory, praxis and policy.

It must be asked who and under what circumstances has the right to part with his or her body, body parts, tissues, and/or cells. These technologies bring back age-old questions and concerns regarding women's right over their bodies, as well as debates around ownership over [11], trade in and leasing of body parts. This becomes crucial because of couples who travel for in-vitro fertilization, several use the oocytes of women or surrogates of the host country. This practice has the potential to be unethical and exploitative as the seemingly free flow of people, capital, goods and services takes place, and is made possible at all, within global relations that are characterised by stark economic inequalities. Not only do unresolved questions of access to these expensive technologies for the majority in third world countries remain, there are far-reaching implications for economically vulnerable women from these countries who participate in ART programmes. This unequal power equation is present not just in cases of foreign clients but also when the recipient individual or couple is from the third world country in question.

## 2. Methodology

This paper presents a brief picture of India's fertility industry with specific focus on its ground-level operation, nature and growth. It aims to explore the industry dimensions of ARTs, by highlighting the macro picture of health care markets and medical tourism in India, the proliferation of the ART industry, market features such as the social imperative to mother, costs, promotion and marketing, unverified claims, inflated success rates, deals and offers, actors and collaborations in the field, and finally, the absence of standards.

This paper presents findings from the research 'Constructing Conceptions: The Mapping of Assisted Reproductive Technologies in India', by Sama, a Delhi-based resource group working on gender, health and rights. This research was conducted from 2008 to 2010 in the three states of Uttar Pradesh, Orissa and Tamil Nadu in India. As part of the research, 43 ART providers and 86 women users, who were undergoing Intra Uterine Insemination (IUI), In Vitro Fertilisation (IVF), or Intra Cytoplasmic Sperm Injection (ICSI), were interviewed. Along with exploring issues of access and regulation, a key objective of this research was to investigate the industry aspects of ARTs in India; this included the inter-linkages between ART clinics in metropolitan cities and those in smaller cities and towns, and the process of 'local globalisation'.

The design of the research was essentially exploratory and qualitative, and sought to document experiences and draw general conclusions based on analyses. The mode of primary data collection involved in-depth interviews, participant observation and focus group discussions. A review of literature, including of promotional materials of clinics, was also undertaken. A team of advisors was instituted to oversee the ethical and methodological aspects of the research. While selecting research sites, a deliberate attempt was made to choose a sample that contained diverse geographical areas with diverse human development indicators, representing different stages of the development of the ART industry in India. Tools prepared for data collection included informed consent forms (in English and local languages), interview schedules, permission letter and field diary. ART providers identified through a mapping exercise were approached directly, and women users were approached through clinics.

This research forms a significant part of Sama's ongoing work on women and technologies, particularly policy advocacy regarding the regulation of the ART industry.

## 3.0 Context

### 3.1 Health Care Market and Medical Tourism in India

The fertility industry in India is an integral part of the country's growing medical tourism industry, which experienced 30% growth in 2000 and 15% growth between 2005-2010 [12]. In 2004 alone, around 150,000 foreigners visited India for treatment [13]. A study by the Confederation of Indian Industry (CII) states that India's potential in this field is so lucrative that it can become a USD 2.3 billion business by 2012. According to one estimate [Research Professor Rupa Chanda, Indian Institute of Management, Bangalore] medical tourism is expected to fetch an impressive USD 4 trillion on a worldwide scale [14]. A World Trade Organization (WTO) study -- conducted in Thailand, Malaysia, Jordan, Singapore and India--concluded that the number of medical travellers to these 5 countries alone was almost 1.3 million persons in the year 2003, collectively earning almost USD 1 billion in treatment costs. Medical travel expenditure in these five countries is growing at the rate of above 20 per cent every year [14]. According to Giuseppe Tattara, a professor of economic policy, in recent years, due to profitability, "more and more investors see the health sector as a good proposition" [12].

The Indian government promotes medical tourism by offering incentives like low interest rates for loans provided to establish hospitals, and subsidized rates for buying drugs, importing equipment, and buying land for clinics. According to the Indian Union Minister for Tourism, for easy access, the Indian government is issuing M (medical) visas to medical tourists and MX visas to accompanying spouses, which are valid for a year [15]. India's National Health Policy (NHP), 2002 states:

"To capitalize on the comparative cost advantage enjoyed by domestic health facilities in the secondary and tertiary sectors, NHP-2002 strongly encourages the providing of such health services on a payment basis to service seekers from overseas. The providers of such services to patients from overseas will be encouraged by extending to their earnings in foreign exchange, all fiscal incentives, including the status of 'deemed exports', which are available to other exporters of goods and services" [16].

Further, the proposed National Health Bill (2009) replaces the provisioning obligations of the state with free access to health care. It thus not only legalises both public private partnerships (PPPs) and medical tourism, but also promises additional state subsidies to the latter through third party payments [17]. Moreover, the General Agreement in Trade in Services (GATS) includes trade in medical services, thus enabling private hospitals treating foreign patients to receive financial incentives; these incentives include the ability to raise capital at low interest rates and eligibility for low import duty on medical equipment [18].

With the combined advantage of low costs and high quality of medical services, India has emerged as a major medical tourism market. Tattara outlines the push and pull factors that make this possible: "Medical tourists are pulled mainly because of reduced costs, the availability of latest medical technologies and a growing compliance with international quality standards, as well as the fact that foreigners are less likely to face language barriers in India. Whereas the cost of treatment in other developed nations, especially in the US, UK, is very high, India can provide quality healthcare at very low cost due to the availability of relatively cheaper but quality manpower, low-priced drugs and other infrastructure" [12] As Qadeer and Reddy assert, medical tourism is an industry that draws on cheaper air fares, internet and communication channels in developing countries, as well as hi-tech super-specialty medical services for people who can afford it - whether foreign or national medical tourists [17]. It also effectively deploys and markets Indian *'exotica'*, and packages health care with other traditional therapies and treatment methods. Services provided include knee joint replacement, bone marrow transplant, bypass surgery, cosmetic surgery, and hip replacement. Assisted Reproductive Technologies form the newest major addition to this list.

### 3.2 Proliferation of the ART Industry

As an integral part of the growing medical tourism industry, the fertility industry is slated to bring in additional revenue of $1-2 billion by 2012 [19]. India is also turning into the surrogacy outsourcing capital of the world; commercial surrogacy and egg donor programmes are fast becoming significant services provided by the fertility industry. Anand [a town in the western state of Gujarat] has become the epicentre of the commercial surrogacy industry in India [19]. While official statistics on the number of surrogacies being arranged in India are not available, anecdotal evidence suggests a sharp increase. According to one estimate:

*The reproductive segment of the Indian medical tourism market is valued at more than $450 million a year and was forecast by the ICMR to be a six billion dollar a year market in 2008. Between 2004 and 2006, the number of websites advertising ART more than quadrupled with marketing heavily geared to foreigners *[20].

Similarly, a newspaper article reports that 50 clinics are added every year to the current 500 IVF clinics in the country, and egg donation is on the rise among women aged 18-35 [21].

In the absence of a national registry, accurate statistics for the number of infertility clinics, or even surrogacies and ART births in the country are not available. A recent article quoted Dr. Thankam Varma, the Medical Director of the Reproductive Medicine and Women's Health Unit at a well known Chennai hospital, as saying that there are over 30,000 ART clinics in the country, while the Indian Council of Medical Research (ICMR) estimates that there are about 3000 ART clinics in India [22]. Nonetheless, ART clinics are no longer concentrated in the metros and big towns, but are also reaching semi-urban areas that otherwise lack even basic civic amenities and essential health care facilities.

Another indicator of the growth of the ART industry in India is the steep rise in the membership of the Indian Society for Assisted Reproduction (ISAR), which was set up in 1997. The number of ISAR members has shot up from 184 in 1997 to over 600 in 2005, which may yet be a conservative estimate [23]. News reports also point to the increasing numbers of foreign clients at ART clinics, and the aggressive promotion strategies adopted by Indian ART providers [24].

## 4.0. Findings: Features of the ART industry

This section will present a summary of the main findings of Sama's research regarding the operationalization of the ART industry in India today. Like any other market, the ART market also deploys common strategies to generate demand, such as offering packages, schemes, and concessions; inflating success rates; and undertaking aggressive advertising through the use of attractively designed websites, brochures, wall advertisements, street hoardings, bus stop signs, and announcements on local television channels [25] The industry is functioning through actors and collaborations at various levels, in an environment where the lack of binding standards or regulation is giving rise to medical malpractice and ethical concerns.

### 4.1. The Logic of Demand and Supply: What women want?

The predominantly private ART industry is characterized by market rhetoric and the language of demand and supply, and takes advantage of the prevailing ideology of patriarchy in society, as well as a collapsing public health system to promote itself. ART providers argue that with infertility "rampant and rising steadily" today, ARTs have become the "need of the hour". They cite higher rates of infections and ensuing complications, particularly in the absence of adequate gynaecological and obstetric services, as factors that contribute to the high infertility in India. Providers thus claim that they are merely responding to the demand of women "desperate" to become mothers [26]. There is an increasing medicalisation and pathologisation of the condition of infertility, with the industry pushing for early medical intervention.

It is not surprising to find that women bear a disproportionate burden of the blame for infertility, including in cases of male factor infertility. Many women internalise this burden. In the event of childlessness, women are routinely harassed (mentally and physically, directly and indirectly, by the community and the family), denied their rightful share in the family's ancestral property, and even abandoned by their husbands [27]. As such, ART providers label these technologies 'pro-women', and as expanding women's reproductive choices. They claim ART is a 'gender-sensitive' technology, and alleviates the suffering that infertile women have to otherwise experience.

The images, language, and slogans used to promote ARTs serve to reinforce the 'tragedy' of childlessness and the sentimentality of childbearing, particularly motherhood, while deliberately ignoring, omitting, or playing down the concerns and complications that come with medical intervention, such as side-effects, efficacy, and costs. While ARTs may 'deliver' women from the social pressure to be mothers, they do not question or challenge this pressure. Further, given the culture of son preference that prevails in Indian society, and India's abysmally low child sex ratio, ARTs raise the fear that the unethical and discriminatory practice of sex selective abortion may be promoted through these technologies [26].

### 4.2 Costs

*"IVF treatment in Singapore is expensive. While treatment in India costs between US$4,000 and US$5,000, more or less, it is at least 1-1/2 times more in Singapore. Besides, Indian doctors have a good reputation as being highly competent and compassionate.(sic)" -*The website of an Indian IVF clinic

The chief reason for India attracting the 'baby business' from other countries is its cost advantage vis-à-vis developed countries. An IVF cycle in the US costs around $20,000 (approximately Rs 9,00,000) as opposed to $2,000 (approximately Rs 90,000) in India. A surrogacy arrangement, including IVF, costs about $11,000 (approximately Rs 5,00,000) in India, while in the US, surrogacy alone, excluding ART charges, costs $15,000 (Rs 6,75,000). In the UK, an IVF cycle costs about £7,000 (Rs 5,00,000 approx) and surrogacy costs about £10,000 (Rs7,00,000 approx) [28]. There is no standardization of costs in the fertility industry, and prices for procedures like IUI, IVF and ICSI vary widely even within India [27].

Undergoing ART procedures involves many hidden costs, such as drugs, travel to the clinic, accommodation near the clinic, loss of work or wages due to repeated clinic visits, etc. When doctors quote treatment prices to users, these costs are often omitted. Nonetheless, despite hidden costs, which could be quite high, the research sample consisted of users from different classes, with several who were willing to push the limits of what they could afford in their quest for a biologically related child.

### 4.3 Promotion

A significant number of the ART clinic websites were found to have exclusive sections devoted to overseas couples. While the amount of space dedicated to this varies, almost all the websites try to seek 'clients' from abroad through promotion of 'medical tourism packages' and incentives, such as discounts and deals on services provided. These generally combine boarding, lodging and other facilities for enjoying the local tourist attractions alongside the ART 'treatment' schedules. Clinics in metropolitan cities like Delhi and Mumbai, where there is large influx of foreign couples and individuals for various ART services, offer IVF cycles in packages that include excursions to nearby tourist attractions like the Taj Mahal, Jaipur palaces, spas in Goa or Kerala etc [25].

*The procedure of IVF does not need any hospitalization it is a day care procedure. You have to visit our clinic for only consultation or Scan or for procedure and that takes not so much of time. .........The total Stay at Delhi will be around 15 to 20 days for a cycle. For stay in Delhi you can contact our Travel Agent ............ All types of accommodation facilities can be managed from budget to Five Star Category, it's depend on your Budget. During stay at Delhi you can also enjoy the City Tour of Delhi, Tour to Taj Mahal, Tour to Jaipur the Pink City and all attraction around Delhi if you like to relax during the procedure (sic)*.

- From the website of an Indian IVF Clinic

*A return air ticket to India from the US costs about US $1000-1500. Your husband can accompany you, or you can hand-carry his frozen sperm in a dry shipper (which you will need to borrow from your local infertility clinic). The clinic is at Bandra, just 20 minutes from the International airport, and is truly in the heart of Bollywood country (Beverley hills of India!)*.

- From the website of a Mumbai-based Fertility Clinic

Almost all website home pages have links that guide the user to services and facilities available and other information related to infertility. Some of the areas that are commonly covered include - a section typically called 'About Us' which provides information about the clinic, facilities and personnel; details about the treatment options for various infertility problems and the services that the clinic provides; IVF success stories and testimonies from clients; success rates, charges/cost of various types of treatment; picture gallery, frequently asked questions, fertility 'myths and facts' and IVF videos [25].

Advertisements carry taglines that promise to 'fulfil dreams', romanticizing what may actually be a long, expensive, unsuccessful and risky medical intervention. Some of these taglines are:

*When nature lets you down, our IVF experts step in and resume the process to bring you the gift of motherhood*.

*They say women make the world go round. How true! It is because they are mothers: The creators and sustainers of every generation*.

*The moment a child is born, the mother is also born. She never existed before*.

The woman existed, but the mother, never. A mother is something absolutely new

With a play of words, a woman's role as a mother is both elevated and venerated to the exclusion of other roles that she performs in society. As such, the linear progression of marriage, motherhood and womanhood is being re/produced, excluding alternative forms of parenthood or voluntary childlessness [25].

### 4.4 (Tall) Claims

Many clinic waiting rooms display photographs of the provider carrying newborn babies, with captions proclaiming "firsts", and other breakthroughs and landmarks. Like any other commercial venture, the ART industry operates in a competitive market environment, which fuels claims of providers to milestones and successes apparently achieved by clinics. These serve to establish the credibility and competitiveness of the clinics, towards attracting users [26].

The city's first test tube baby arrives

*In a short span of 3 years, we now delivered about 300 babies using the state-of-the-art facilities*.

*Unique test tube baby centre, which is the first in Orissa, and has delivered the 1st IVF and ICSI baby in Orissa*.

### 4.5 Inflated Success Rates

Inflating success rates to attract consumers is also common in the ART industry. In order to promote their services and expand their clientele, ART providers quote success rates that are often exaggerated or unclear and misleading [26].

Success rate can be reported in various ways by clinics. Many report the embryo implantation or pregnancy rate as the success rate; these are higher than the live birth rate because a pregnancy may end in miscarriage, or induced abortion, or stillbirth. Clinics rarely quoted the take-home-baby or live birth rate as the success rate, and users are generally unaware of the difference. Moreover, the success rates quoted by clinics are nearly never substantiated on the basis of the number of users or the time period with regard to which they were calculated. This makes it difficult to discern the extent of the 'success' denoted by stand-alone figures and percentages. Further, success rates vary with the type of procedure used, whether IUI, IVF, or ICSI. Clinics, however, often quote one success rate, without any qualification indicating the specific procedure to which the rate refers [26].

Our pregnancy rates at 65-70% are among the highest in the world

*Today we have a success rate of 40-50% per treatment cycle*.

*The success rate of ICSI & Test Tube Baby is 50% to 60% comparison to best Laboratory in the World*.

These rates quoted by clinics exceed the internationally accepted success rates by a large margin, thus putting into question their authenticity. These rates were quoted by providers themselves, and were found in the promotional material of clinics. The 'success stories' too are magnified and over played.

### 4.6 Package Deals, Schemes, Concessions and Camps

The idea behind offering a 'package deal' is the same as in any other service - encouraging/luring the user to purchase more services or products, by projecting their combined cost as lower than the sum of their individual costs, thus making the deal seem economical. Packages in IVF gained popularity with the rise in medical tourism.

Clinics also offer schemes such as 'shared risk scheme', 'egg sharing scheme', and 'money back guarantee scheme', which reduce the treatment costs in ARTs [26]. In the egg-sharing scheme, a woman undergoing IVF shares her eggs with another woman undergoing IVF in lieu of a reduction in the cost of her IVF cycles. This is becoming common even in clinics in smaller towns and cities.

While packages and schemes benefit both the user and the provider of Arts, concessions, another feature, may be given at random by the provider to specific users. These are expressions of the providers' benevolence, which in turn earn them goodwill and help to spread word about their clinic [26].

Yet another feature of the ART market is the organization of infertility camps by ART clinics, in line with camps for free health check ups, dental check ups, eye check ups etc that have been common in India. Now, ARTs have jumped on the bandwagon of this popular recruitment strategy. One clinic in UP held 'free infertility and IVF consultation camps' and provided special discounts on tests and procedures of IUI and IVF, if needed. These camps may be advertised in clinic websites, or local newspapers [26].

### 4.7. Actors and Collaborations in the ART Industry

ART clinics are not the only players in the business of promoting 'reproductive tourism' in India. Other emerging players include a wide array of organizations catering to clientele both at the national and international levels. These range from ART consultants, medical tour operators, surrogacy agents, the hospitality industry, and tourism departments to other organizations specializing in medical tourism promotion.

Consultancy agencies like Indian Med Guru and Forerunners Healthcare Consultants cater exclusively to international users. For example, Indian Med Guru defines itself as "... a consultancy for infertility treatment and artificial reproductive techniques, in India, which addresses the need of international patients" http://www.indianmedguru.com. Agencies like Trivector Scientific International and ART Associates provide "expertise" to ART clinics to upgrade their facilities and technical capacity for a more effective marketing of their services internationally. Such groups either specialise in a particular service or follow an approach of 'all-under-one-roof'. They tend to present their services as containing an element of social work/service. Royal Medical Tours (Mumbai) Pvt. Ltd. promotes health packages designed along the lines of regular tour packages, except with the added dimension of helping their clients obtain medical treatment [25].

In another interesting phenomenon emerging in the ART market today; joint collaborations are coming up, wherein ART clinics in India have tied up with international hospitals and agencies to solicit clients globally. Some of these companies are headquartered in the United States or in other countries, from where the clients are sourced. Planet Hospital (PH), a medical tourism agency with headquarters in California, has an exclusive surrogacy arrangement with Dr. Gautam Allahbadia, the Mumbai-based director of Rotunda--The Center for Human Reproduction. PH's client base is primarily American, but also consists of EU citizens and persons of Indian origin living in the United States. The company receives 15 to 20 inquiries per day regarding surrogacy [26]. Rudy Rupak, co-founder and president of PH, said he expected to send at least 100 couples to India in 2008 for surrogacy, up from 25 in 2007, the first year he started offering the service. "Every time there is a success story, hundreds of inquiries follow," he asserted [29].

Reverse tourism is seen to occur in egg donation, with companies bringing in women from first world countries to donate their eggs as well as travel in India. Florida based Proactive Family Solutions (PFS) is one such subsidiary that recruits intended parents and egg donors. PFS provides intended parents with a pool of potential egg donors based on the client's criteria, which typically include hair and eye colour, and education level. The company takes care of everything the egg donor might need in India, and even accommodates the egg donor's academic schedule, arranging for her to travel to India during school breaks in case she is a student. The egg donor may also bring one person to accompany her from the US [26].

Surrogacy centres and hostels that house surrogate women for the duration of their pregnancy are also emerging. New and multiple actors, like surrogacy agents, are now part of this industry. One such surrogacy agent claimed that he was able to match, on an average, one couple with a woman willing to be a surrogate every month. In surrogacy hostels like the one in Anand, surrogate mothers are carefully chosen, and are cared for with nutritional and medical support, which-- given their typically low socio-economic backgrounds-- is ironically probably what they missed when they gave birth to their own children[26].

### 4.8 Market Without Rules

Can the standard competitive market model, with free market principles, be considered adequate for the health care sector? If we consider the ethical and physical hazards involved in malpractices, the unequal access to information between the users and providers, as well as the uncertainty of outcome of procedures, the answer must be no. Some of the medical malpractices that the study revealed were as follows:

• There was a lack of standardization in treatment protocol, such as the number of cycles, gap between cycles, etc. This paves the way for the exploitation of users, both physically and economically. This was seen both within and across procedures like IUI, IVF, and ICSI.

• Not only were procedural costs for IUI and IVF found to vary widely, even the costs of the drugs used were found to be disparate. This variation was found not only across the three research states of Tamil Nadu, Uttar Pradesh and Orissa, but also between clinics in the same state.

• Side effects of the procedures, such as ectopic pregnancies, and the potentially fatal Ovarian Hyper Stimulation Syndrome (OHSS) were under-represented to users. Multiple births, which carry serious risks to the health of the mother and the children, were celebrated by clinics as an achievement, and widely advertised.

• Users had inadequate and piecemeal information about their treatment, including procedures, drugs, side effects and overall costs. Counselling, which should be a mandatory, comprehensive and sustained process, was found to be a one-off information-giving exercise, if at all. This was in marked contradiction to the notion of "informed" choices that consumers are expected to make in a competitive market.

• The process of obtaining informed consent was treated as a mere formality, with little attention being paid to the content of the informed consent form. In several instances, no form had been signed, or forms had been signed without being read, or by proxy.

• Practices like sex selection, multiple embryo implantation and even the inducement of pregnancy in postmenopausal women, are common. Given the present climate for son preference, ARTs have the (unchecked) potential to encourage pronatalist eugenics and attitudes to design one's own child (preferably male). Though the Preconception and Pre-natal Diagnostic Techniques (Prohibition of Sex Selection), PCPNDT Act, (1994) 2003 prohibits sex selection before and after conception, and regulates the use of new reproductive technologies, evidence of the use of ARTs for sex selection was found in the research.

The absence of any legally binding regulatory mechanism is exploited to the maximum extent possible by providers. The only document guiding the conduct of ART clinics in India at present is the 'National Guidelines on Regulation, Supervision and Accreditation of ART clinics in India', released by the Indian Council of Medical Research (ICMR). This is non-binding in nature. In 2008, the Ministry of Health and Family Welfare (MOHFW) and the ICMR released the ART (Regulation) Bill and Rules 2008. While this was a welcome step towards regulation, concerns regarding the health and rights of women users were raised by civil society groups. Sama prepared a policy brief for parliamentarians in 2009, critiquing problematic provisions of the draft bill. Since then, the ICMR has released another revised version, the Draft ART (Regulation) Bill and Rules 2010. The 2010 draft has taken some of the civil society concerns into consideration, while excluding several others. As such, many ethical issues that are emerging out of unrestrained spread of the technologies remain. Thus, while regulation of ARTs is desirable, proposed legislation must centre-stage the rights of the most vulnerable, which in this case are the women users, surrogates, and the children born with ARTs.

## 5. Conclusion

*"Although, the market is the primary motor of globalization, its implications are not limited to the commercial arena alone. In the field of biological reproduction, globalization - understood as the rapid growth of global capitalism - has brought in its wake an extension of consumer culture creating 'new regimes of consumption". -*Jyostna Agnihotri Gupta [30]

At the core of the 'business' of IVF is, of course, reproduction, increasingly seen as a professionalized and commercialized domain, wherein women's procreative capacity can be tested, stimulated, broken down, transferred, frozen, bought and sold. It is this convergence of professional, technological, and commercial "management" of reproduction that has generated widespread public debate. The fact that infertility treatment today is most commonly associated with business even in government documents is worrying enough, but the problem gets further exacerbated and complicated by the fact that there is a overlap between clinical IVF practice (the 'marketplace' where services are sold and consumed) and sites where new fertilization technologies are developed and tested [31] As such, the after-life of left-over embryos and gametes from IVF, used in stem cell research, raises as yet under-explored ethical concerns such as consent and ownership,

ARTs in the Indian context have proliferated with rapid pace and have become a booming market within the already booming medical tourism industry. The implications of ART use include, but are not limited to, deterioration of health, with a direct impact on the social and physical functioning of individuals, increased health risks to children born with ARTs, psychological problems and high stress levels, geographical and social relocation, strained sexual relations, disruption of work and daily routines, and financial instability. Yet, the desire for a biological child is strong, and for women in particular, the alternative to ARTs--the stigma, even violence, of a childless life--may be no alternative at all. Further, services for infertility care, including basic screening facilities, are conspicuous by their absence in the public health system in India; this includes health infrastructure for addressing preventive and secondary causes of infertility, which can be combated at a preliminary stage. This raises the question of equity in reproductive health and rights: if the right of the infertile, and of LGBTQ individuals and couples, to have biologically related children is a legitimate reproductive right, then what of their poorer counterparts? The movement of babies, reproductive body parts and women's reproductive labour and care work -- as nannies, egg donors and surrogates-- has led to the "globalization of motherhood" [32]. As is obvious, this impacts women who mother, and women who enable other women to mother. While this sets the stage for the market to flourish, drawing on capitalist principles of profiteering and deployed to cash in on patriarchal values, this market is also where, as Betsy Hartmann says, "exploitation and opportunity are bound and wound up in one" [33]. Perhaps the pertinent question then is: how can we ensure that the crossing of geographic and 'biological' boundaries does not become a crossing of ethical boundaries?

## Competing interests

The authors declare that they have no competing interests.

## Authors' contributions

SN and VM conceptualized and drafted the manuscript. AS revised the manuscript. All authors were part of the research team, and read and approved the final manuscript.
